# Of Lymph Nodes and CLL Cells: Deciphering the Role of CCR7 in the Pathogenesis of CLL and Understanding Its Potential as Therapeutic Target

**DOI:** 10.3389/fimmu.2021.662866

**Published:** 2021-03-24

**Authors:** Carlos Cuesta-Mateos, Jennifer R. Brown, Fernando Terrón, Cecilia Muñoz-Calleja

**Affiliations:** ^1^ Immunology Department, Hospital Universitario de La Princesa, Instituto de Investigación Sanitaria- Instituto de La Princesa (IIS-IP), Madrid, Spain; ^2^ IMMED S.L., Immunological and Medicinal Products, Madrid, Spain; ^3^ Catapult Therapeutics BV, Lelystad, Netherlands; ^4^ Chronic Lymphocytic Leukemia (CLL) Center, Department of Medical Oncology, Dana-Farber Cancer Institute, Boston, MA, United States; ^5^ School of Medicine, Universidad Autónoma de Madrid, Madrid, Spain

**Keywords:** CCR7, CLL (chronic lymphocytic leukemia), lymph node, pathophysiology, immunotherapy

## Abstract

The lymph node (LN) is an essential tissue for achieving effective immune responses but it is also critical in the pathogenesis of chronic lymphocytic leukemia (CLL). Within the multitude of signaling pathways aberrantly regulated in CLL the homeostatic axis composed by the chemokine receptor CCR7 and its ligands is the main driver for directing immune cells to home into the LN. In this literature review, we address the roles of CCR7 in the pathophysiology of CLL, and how this chemokine receptor is of critical importance to develop more rational and effective therapies for this malignancy.

## Introduction

Lymph nodes (LN) function as a major immunological hub, essential for immune homeostasis and generation of effective immune responses (1). Yet LNs are also a fundamental tissue in the development, progression and treatment failure of several mature lymphomas/leukemias, especially for chronic lymphocytic leukemia (CLL) (2–6). In recent years growing evidence suggests that cell trafficking orchestrated by the chemokine receptor CCR7 plays a critical role in the pathophysiology of CLL. LN stromal cells secrete CCR7 ligands generating powerful chemotactic gradients that attract CLL cells into the microenvironment, where a diversity of cells, soluble factors, and matrix proteins facilitate survival and proliferative cues, thus promoting disease progression and preventing spontaneous or drug-induced apoptosis of leukemic cells. In this literature review we provide in depth insight into how CCR7-mediated functions contribute to CLL pathogenesis, and how this chemokine receptor may be a critical potential therapeutic target in CLL.

## CLL

With an age-adjusted incidence of 4.3/100000 inhabitants in the United States, CLL is the most common type of leukemia in Western countries. More than 20000 newly diagnosed cases and ~4500 deaths per year are currently estimated (7). CLL is characterized by the clonal proliferation and accumulation of mature, typically CD5-positive B cells within the peripheral blood (PB), bone marrow (BM), LNs, and spleen. Despite a remarkable phenotypic and cytological homogeneity, CLL is characterized by extremely variable clinical course related to different prognostic factors including the mutational status of the immunoglobulin heavy-chain variable region (IGHV) (8–10), expression of very late antigen 4 (VLA-4), CD38 and zeta-associated-protein 70 (ZAP-70) markers (11, 12), and specific cytogenetic alterations including the most common and early event of deletion of the long arm of chromosome 13 [*del(13q14*)] and other alterations that occur later in the course of disease and predict worse outcome such as *del(11q)*, and *del(17p)* (13–15). In recent years, whole-genome sequencing has uncovered novel recurrent somatic gene mutations that occur in CLL cells in parallel to the above-mentioned structural genomic aberrations. Of these, mutations affecting the genes *NOTCH1*, *p53*, ataxia-telangiectasia-mutated *(ATM)*, and splicing factor 3b subunit 1 (*SF3B1)* seem to be more common, with a long tail of less common but nonetheless recurrent driver mutations (16–19).

Until very recently, chemoimmunotherapy, the combination of monoclonal antibodies (mAb) against CD20 with chemotherapy, was the most effective therapeutic approach in CLL. In particular, standard therapy with the combination of FCR (fludarabine, cyclophosphamide, rituximab) was shown to prolong both progression-free survival and overall survival (OS) in CLL (20) and to result in long-term remission in patients with mutated IGHV. Response in patients with TP53 aberrant disease was poor however, and patients with unmutated IGHV generally showed continuous relapse even after initial deep response, including undetectable minimal residual disease (MRD) responses. The development of Bruton´s tyrosine kinase (BTK) inhibitors in particular as well as more recently the B cell lymphoma 2 protein (BCL-2) inhibitor venetoclax has led to more effective therapy particularly for higher risk disease (21, 22). Phosphatidylinositol 3 kinase (PI3K) inhibitors also have significant activity but have been hampered by toxicity. Despite the efficacy of these drugs, continuous therapy is required with the B cell receptor (BCR) pathway inhibitors leading to toxicity and cost, as well as increasing relapse over time. The venetoclax regimens have been developed to be time-limited, and follow-up is still too short to know the durability in different disease groups. It is clear that patients who do not achieve undetectable MRD with a venetoclax regimen have steady relapse and constitute a group with unmet need. All higher risk patient groups, particularly those with p53 aberrant disease, complex karyotype and even unmutated IGHV, all have higher risk of relapse and still have significant unmet medical need for additional treatment strategies (21–23).

A hallmark of the pathophysiology of CLL is that blood circulating leukemia cells are mainly in a G_0_/G_1_ cell cycle–arrested phase, whereas CLL cells within LN are proliferating and hence promote disease progression (2, 4). In this scenario, CLL is seen as a dynamic neoplasm comprising leukemic cells that multiply and die at measurable rates (24). However, and at variance with other hematologic malignancies, CLL proliferation rates are relatively low and cell accumulation is the result of an abnormally prolonged survival rather than uncontrolled proliferation (25). Indeed, intrinsic defects in the apoptotic machinery such as overexpression of BCL-2 and myeloid-cell leukemia 1 (MCL-1) anti-apoptotic members, or impaired expression of pro-apoptotic members (Bax and Bak), and extrinsic factors consisting mainly of stromal cell–derived cytokines and chemokines (e.g. CXCL12), provide survival cues during which tumor cells transit through lymphoid tissues and tilt the balance toward prolonged lifespan of CLL B cells (6, 26).

### CCR7 and Its Ligands

The homeostatic chemokine receptor CCR7 was identified in the 1990s as the first lymphocyte specific G-protein coupled receptor (GPCR) (27–29). Also known as Epstein–Barr virus-induced gene 1 (EBI1), Burkitt’s lymphoma receptor 2 (BLR2), or CD197, this 378 amino acid protein is encoded by a gene located on human chromosome 17q12-21.2 (28). CCR7 is expressed by various immune cells including double negative (DN) and single positive (SP) thymocytes, naïve, central memory and regulatory T cells (T_N_, T_CM_, T_REG_), naïve B cells, CD56^+^CD16^-^ regulatory natural killer (NK) cells, and (semi-)mature dendritic cells (DCs) (30–32). In addition, CCR7 expression has been found in different non-immune cells, most notably in various malignancies (32). Generally, mentioned T cells subsets and mature B cells constitutively express CCR7 whereas NK cells and DCs acquire CCR7 expression upon encountering a pathogen (30). In both homeostasis and cancer, CCR7 but not other receptors, specifically drives cell homing into LN and other secondary lymphoid organs (SLO) (33–35). This GPCR orchestrates: cell trafficking, firm arrest to endothelium, extravasation, positioning within SLO, activation, and egress upon binding two cognate ligands, the chemokines CCL19 (aka ELC or MIP-3β) and CCL21 (aka SLC or 6CK), constitutively expressed by stroma cells in SLOs and present on lymphatic vessels, high-endothelial venules (HEVs), and T zones. In addition, CCL21 is produced by lymphatic endothelial cells (30, 31, 36). Both chemokines share only 32% sequence homology and are structurally and functionally distinct (37). Indeed, both molecules differ in length with CCL21 encoding a 37 aa long C-terminal tail extension, that is lacking in CCL19, and which is rich in positively charged (basic) residues. This tail, which can be proteolytically cleaved, confers high affinity for negatively charged molecules of the extracellular matrix (ECM), including glycosaminoglycans (GAGs), therefore the lack of such C-terminal basic extension in CCL19, and in CCL21 tail-less form impairs its ability to form haptotactic gradients (36, 38–40).

### The LN in CLL

Upon immune activation, reactive LN acquire a characteristic structure (1, 41). Three main cellular compartments are easily distinguishable: the cortex, the paracortex and the medulla. The cortical area contains lymphoid follicles composed mostly of B cells (B zone). In reactive LN, the primary follicles evolve to secondary follicles, made up of a germinal center (GC) and surrounding mantle zone. In paracortical areas (or T zone), T cells predominate and are mixed with interdigitating DCs, plasma cells and “tingible body” macrophages (TBM). Finally, the medulla consists of the medullary cords, which contain lymphocytes, plasma cells, and macrophages, and the medullary sinuses, contiguous with the efferent lymphatics, and contain lymph, macrophages, plasma cells, and mast cells.

**Figure 1:**
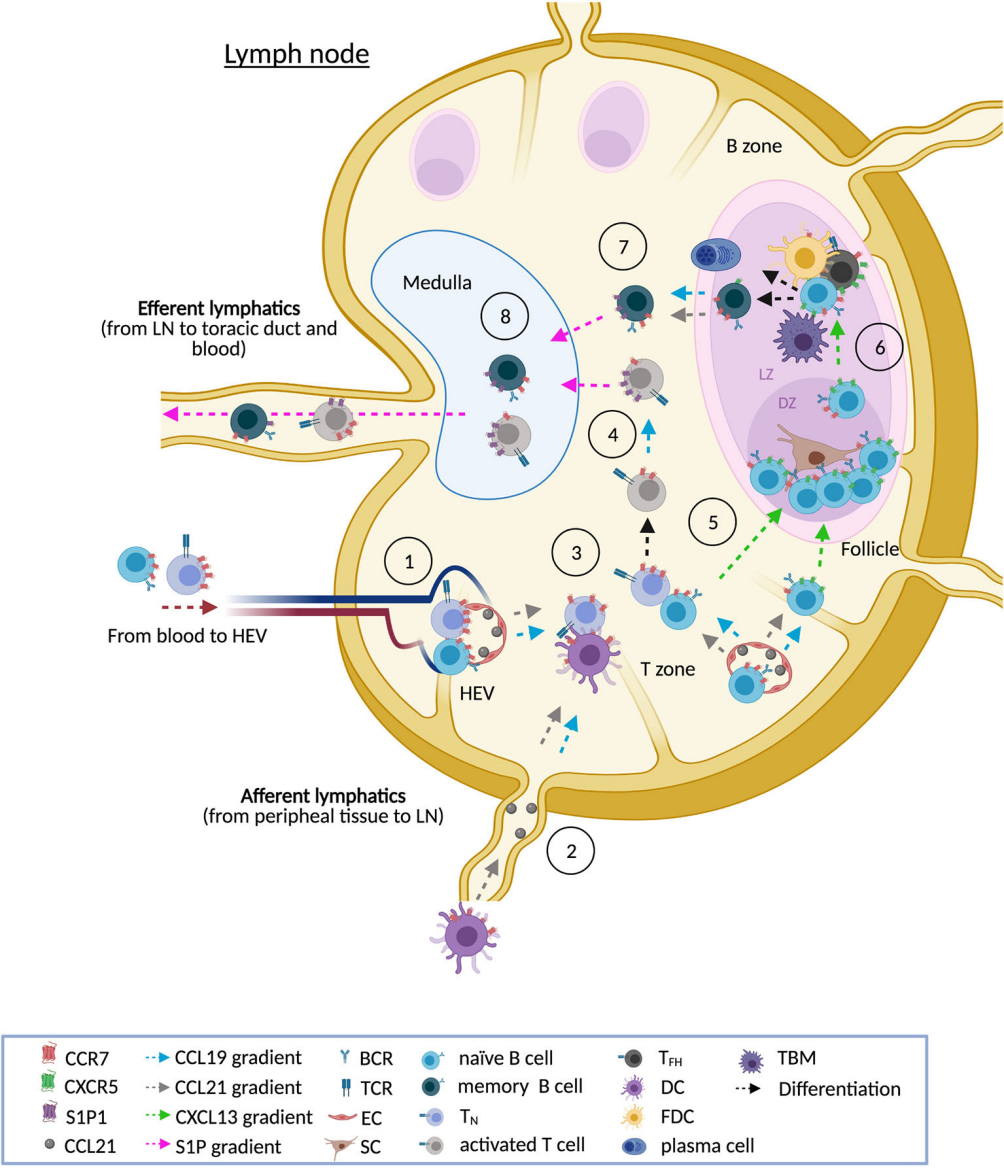
CCR7 and the reactive LN. In homeostasis, normal LN show three main cellular compartments: the cortex (B zone), the paracortex (T zone) and the medulla. Upon antigen stimulation, the primary follicles evolve to secondary follicles, made up of a germinal center (GC) and surrounding mantle zone. In reactive LN, CCR7 is necessary for the entry of naïve B cells, naïve T cells (T_N_), regulatory T cells (T_REG_, not shown), central memory T cells (T_CM_, not shown), and dendritic cells (DCs). CCR7 guides lymphocyte homing through high endothelial venules (HEVs) in the paracortex [1] whereas DCs preferentially use afferent lymphatics [2]. CCR7 also drives interstitial migration of these immune subsets in the T zone facilitating, for instance, the interaction of T_N_ with antigen presenting cells such as B cells and DCs [3]. Upon activation, T cells are directed to the medulla following CCL19 gradients. CCL19 signaling also induces CCR7 internalization and the up-regulation of the egressing receptor S1P1. The balance between CCR7 and S1P1 is needed for the movement of activated T cells from the T zone to the medulla [4]. Similarly, a fine-tuned balance between CCR7 and CXCR5 allows the migration of activated B cells through the T zone and the follicle. In a first step, CCR7 is down-modulated while a concomitant up-regulation of CXCR5 allows activated B cells to enter into the follicle [5]. In reactive follicles, fully developed GC are polarized into two regions clearly differentiated: the dark zone (DZ) and the light zone (LZ). Although GC B cells re-express CCR7, migration of GC B cells between both regions relies on the CXCR5-CXCL13 axis [6]. In the DZ, GC B cells (centroblasts) interact with stromal cells, proliferate (clonal expansion) and undergo somatic hypermutation on the immunoglobulin genes. In the LZ, hypermutated resting GC B cells (centrocytes) interact with a dense network of CXCL13^hi^ follicular dendritic cells (FDCs) and CXCR5^hi^ follicular helper T cells (T_FH_). FDCs display antigen and secrete cytokines and chemokines (CXCL13) that attract B cells and T_FH_ to the GC. T_FH_ are specialized CD4^+^ PD-1^+^ T cells that express BCL-6 and secrete cytokines that promote B cell proliferation and differentiation. T_FH_ deliver survival signals to GC B cells through a number of different pathways, including CD40-CD40L, PD1-PDL1, and IL-21. The pro-survival signals from T_FH_ counteract pro-apoptotic signals from the FAS-FASL pathway. Crosstalk of centrocytes with FDCs and T_HF_ allows the class-switch recombination and the selection of B cells. Centrocytes with the appropriate antigen affinity are selected to become memory B cells or antibody secreting plasma cells. The centrocytes that are not selected undergo apoptosis and are removed by tingible-body macrophages (TBM). Expression of CCR7 allows memory B cells to exit from follicles back to the T zone and, from there, to the medulla [7]. S1P1-expressing T cells and B cells move towards the efferent vessels following S1P gradients [8]. Notation: this scheme shows the main cell types in a reactive LN and in the GC, however in these complex tissues participate additional subtypes not listed here that can be further reviewed elsewhere (1, 30, 31, 41).

In homeostasis, reactive LN is an important site for B cell-Ag interaction, initiating and supporting multiple processes including heavy immunoglobulin chain class switching, somatic hypermutation and proliferation (2). These processes take place on the GC, where B cells interact with helper follicular T cells (T_HF_) and follicular dendritic cells (FDC), that serve to increase the variety of Ag binding sites available for clonal selection (2). In this normal tissue, CCR7 is expressed in B cell and T cell zones with a marked positivity on centrocytes and centroblasts in secondary follicle GCs (42). Additional aspects related to reactive tissue structure and CCR7 expression are disclosed in **Figure 1**.

In CLL, the LN frequently shows a pattern of architectural effacement by homogeneous diffuse sheets of small CLL lymphocytes, often obliterating the normal nodal tissue, and exhibits small, if any, residual GCs. In many cases, neoplastic cells with increased mitotic activity form scattered foci that resemble germinal centers called pseudofollicles or proliferation centers (PCs) which are enriched with prolymphocytes and paraimmunoblasts (4, 43–46). In CLL patients, the PCs are much more evident in LN than in the spleen and the BM; they occur in 88% of LN biopsies. Moreover, PCs are hallmark features in the LN of patients with CLL as they are not observed in other B-cell tumors (4).

Unlike reactive GCs, the leukemic cells of PCs lack a GC B-cell phenotype, but may contain increased CD40L^+^CD4^+^ T_H_ cells and in some cases a fine network of DCs, suggesting that PCs likely do not arise secondary to colonization of GCs by tumor cells (44). In contrast, the PCs tend to have less well-defined borders without mantle zones, lack polarity, and do not contain tingible body macrophages, allowing for easy discrimination from GC (44).

### CCR7 Over-Expression in CLL

A large series of studies have reported that the CLL cells of almost all patients express high surface levels of CCR7, where a distinct, intense positive peak consisting of > 96% of positive cells is usually observed by flow cytometry (14, 42, 47–66). This expression of CCR7 in CLL is abnormally high when compared to the corresponding normal CD5^+^ B-cell population or pan-B cells. The CCR7 expression is consistently observed in both resting and proliferative compartments; in different groups of CLL patients regardless of the stage at diagnosis; unfavorable flow cytometry (CD38, ZAP-70, VLA-4) or genetic (*IGHV* mutated/un-mutated status, *p53* mutated/deleted, *ATM* mutated/deleted, trisomy 12, *NOTCH1* mutations) prognostic markers; previous treatments or the presence of relapsed or refractory (r/r) disease to different standard-of-care (SOC) therapies (**Table 1**). Similarly, in CLL nodes, a strong CCR7 positivity is seen in small lymphocytes and paraimmunoblasts of PCs (42), and this expression did not differ between matched LN and BM biopsy samples, hence, CCR7 levels remains high on CLL cells following their accumulation in SLOs and BM.

**Table 1:** Publications reporting CCR7 expression in CLL cells.

Reference	Samples	Technique	Main findings (related to CCR7 expression)
**Klein et al.** (47)	**CLL** (PB; n=10) **HD** (tonsils; memory B cells; n=5)	GEP	3.3 fold-change *CCR7* gene expression in CLL (M/UM) vs memory B cells.
**Till et al.** (48)	**CLL** (PB; n=30)	FACS TEM	CCR7 expression regardless of B/R-stage, CD38, M/UM, UT/T. Expression correlated with nodal involvement. TEM (CCL21) correlated with clinical lymphadenopathy but not with VH mutations or CD38.
**Lopez-Giral et al.** (49)	**CLL** (PB, BM, LN; n=79) **CD5^-^ CLL** (PB, BM; n=12) **HD** (PB, BM, tonsils; n=10)	FACS Chemotaxis	CCR7 is highly expressed in CLL vs HD (4.4 fold change CLL vs CD5^+^ B cells; 14.3 fold-change CLL vs CD10^+^ B cells; 10 fold-change CLL vs pan-B cells). CCR7 expression regardless of the B/R-stage, CD38, M/UM, UT/T. CLL preferential migration towards CCL19/CCL21 vs CXCL12 or CXCL13. Migration correlated with nodal involvement but not with CD38 or VH mutations.
**Wong et al.** (50)	**CLL** (PB; n=21) **SLL** (LN; n=9) **HD** (PB; n=20)	FACS	CCR7 is highly expressed in CLL vs HD. Expression in SLL similar to HD.
**Ghobrial et al.** (51)	**CLL** (PB and matched LN; n=33 and 5). **SLL** (PB and matched LN; n=12 and 12). **HD** (PB and reactive LN; n=5 and 3)	FACS	CLL with a marked increase in CCR7 expression (6.6 fold change PB CLL vs normal B cells; 2.4 fold change in CLL vs reactive LN). No significant difference in the expression of CCR7 between LN of patients with and without peripheral lymphocytosis. CCR7 expression correlated with Rai stages and lymphadenopathy but not with VH mutations, CD38, hemoglobin, WBC count, platelet count, and chemotherapy use.
**Rodriguez et al.** (67)	**CLL** (PB and LN; n=41) **HD** (reactive LN; n=4)	GEP	*CCR7* up-regulated in CLL LN vs reactive nodes
**Alfonso-Pérez et al.** (52)	**CLL** (PB/BM; n=11) **HD** (PB; n=6)	FACS	CCR7 expression is higher in CLL than in CD3^+^ T cells from CLL patients (3.4 fold change); normal CD19^+^ pan-B cells (2.6 fold change), normal CD3^+^ pan-T cells (2.4 fold change), and DCs (3.4 fold change). Expression is found regardless of the stage, WBC counts, VH mutations, cytogenetic, T/UT, CD38 or ZAP-70ZAP-70.
**Chunsong et al.** (68)	**CLL** (PB; n=16) **HD** (PB; CB; n=8)	FACS	CCR7 expression in CLL is higher than in CD19^+^CD5^-^ cells (6.5 fold change), but lower than CD23^+^CD5^+^ cord blood cells (1.2 fold change).
**Richardson et al.** (53)	**CLL** (PB; n=38) **HD** (PB; n=6)	FACS Chemotaxis	CCR7 expression is higher in clones with ZAP-70ZAP-70 expression than ZAP-70ZAP-70-negative cells (1.5 fold change). CLL PB cells expressed significantly higher CCR7 surface levels (5- to 7-fold increase) when compared with control PB B cells. ZAP-70ZAP-70^+^ CLL cells are more responsive to CCL19/CCL21 than ZAP-70ZAP-70^-^ cells but are equally responsive to CXCL12 CCR7 expression is found regardless of the Rai stages, VH mutations, CD38, hemoglobin, WBC count, cytogenetic, and UT/T.
**Ticchioni et al.** (69)	**CLL** (PB; n=26)	FACS	CCR7 is expressed in CLL samples regardless VH mutations, clinical stage, ZAP-70ZAP-70, or CD38.
**Redondo-Muñoz et al.** (70)	**CLL** (PB; n=6)	FACS	CCR7 is expressed in CLL samples.
**Enjuanes et al.** (71)	**CLL** (n=692) **HD** (n=738)	GEP/genotyping	One SNPs (rs3136687, intron 1) was associated with CLL development. No differences in the expression levels were observed for this CCR7 variant.
**Trinidad et al.** (72)	**CLL** (PB/BM; n=13)	FACS	CCR7 is expressed in CLL samples. Similar high levels were found in patients with and without nodal involvement (n= 4 and 9).
**Calissano et al.** (54)	**CLL** (PB; n=13)	FACS	Similar CCR7 levels between resting (CD38^-^) and proliferating (CD38^+^Ki67^+^) clones.
**Catusse et al.** (73)	**CLL** (PB; n=4) **HD** (PB; n=4)	FACS	CCR7 expression is higher in CLL than normal B cells (12.5 fold change).
**Cuesta-Mateos et al.** (63)	**CLL** (PB, BM; n=20) **HD** (PB; n=15)	FACS	CCR7 expression is higher in CLL than normal B cells (3 fold change).
**Calissano et al.** (56)	**CLL** (PB; n=20)	GEP FACS	Among 1299 genes differentially expressed between the proliferating (CXCR4^dim^CD5^bright^) and resting (CXCR4^bright^CD5^dim^) CLL compartments, CCR7 was not included. Immunophenotype of both fractions showed high levels of CCR7 expression although slightly greater levels were seen in proliferating cells in terms of percent positive cells and/or MFI.
**Bryson et al.** (57)	**CLL** (PB, BM; n=24)	FACS	CCR7 expression is found in all samples.
**Zuchetto et al.** (58)	**CLL** (PB, n=49)	FACS	CCR7 with a higher expression in the UM CLL group. CCR7 is proposed to be included as a routine diagnostic/prognostic marker by flow cytometry.
**Calpe et al.** (59)	**CLL** (PB, n=40)	FACS, Chemotaxis	Expression of CCR7 was significantly higher in CLL cells with high ZAP-70ZAP-70 expression within the same patient. CLL cells migrating toward CCL21 had a significantly higher percentage of ZAP-70ZAP-70–positive cells. ZAP-70ZAP-70 signaling induces the expression of CCR7 in B cells through ERK1/2 phosphorylation.
**Capitani et al.** (60)	**CLL** (PB; n=57) **HD** (Buffy coat; n=8)	GEP	Higher CCR7 mRNA in CLL than HD; higher content in UM than M CLL. P66Shc controls CCR7 expression in CLL cells.
**De Rooij et al.** (61)	**CLL** (n=5)	FACS Chemotaxis Adhesion	CCR7 expression is higher in CLL than normal B cells (5.5 fold change). CCR7 expression, migration and adhesion are impacted by ibrutinib.
**Somovilla-Crespo et al.** (62)	**CLL** (PB, BM; n=79) **CD5^-^ CLL** (PB, BM; n=5) **HD** (PB; n=4)	FACS	CCR7 expression is higher in CLL than normal B cells (10 fold change). CCR7 expression is higher in CD5^-^ CLL than normal B cells (5 fold change).
**Girbl et al.** (74)	**CLL** (n=8)	FACS	CD40L stimulation of CLL cells induces an activated phenotype with augmented CCR7 expression and reduced motility on immobilized HA/CCL21 as a consequence of CD44v-HA strong interactions.
**Eagle et al.** (75)	**CLL** (PB; n=18)	GEP Migration	No differential gene expression between M and UM CLL. No significant differences were seen in migration towards CCL21 in M and UM cells.
**Cuesta-Mateos et al.** (63)	**CLL** (PB; n=23) **HD** (PB; n=6)	FACS	CCR7 expression is higher in CLL than normal B cells (5 fold change). CCR7 is highly expressed on CLL cells regardless clinical stage, adverse cytogenetic prognostic factors or previous treatments.
**Patrussi et al.** (42)	**CLL** (PB; n=52) **HD** (Buffy coat; n=10)	GEP FACS	CCR7 mRNA levels are higher in CLL (M and UM) than normal B cells (2.6 and 3.6 fold change). CCR7surface expression levels are higher in CLL (M and UM) than normal B cells (3 fold change). Total cell content is 4 and 5 times higher in CLL cells than in normal B cells. UM CLL cells showed a preferential binding and migration towards CCL21.
**Ganghammer et al.** (14)	**CLL** (PB; n=85)	FACS	CCR7 surface levels high in CLL cases regardless the presence/absence of CD49d and/or tri12. CCR7 surface levels (MIFR) correlated with CD49d.
**Haerzschel et al.** (64)	**CLL** (PB; n=19) **HD** (PB; n=5)	FACS Chemotaxis	CCR7 expression is not affected by IgM and IgD stimulation. CCR7 surface levels higher in CLL cells than in normal B cells. IgM-stimulated CLL cells retained chemotaxis towards CCL21 whereas Ig-D stimulated CLL cells showed reduced response towards the same chemokine.
**Arruga et al.** (65)	**CLL** (PB; n=39)	GEP Chemotaxis	CCR7 expression (mRNA) is similar between NOTCH1-M and-UM CLL clones. CCL19-induced migration is more efficient in NOTCH1-M CLL samples.
**Wolf et al.** (76)	**CLL** (PB; n=29) **HD** (PB; n=18)	GEP	Correlation between CCR7 and NFATC1 expression (mRNA) CCR7 is significantly overexpressed in CLL cells compared to healthy donor samples (3 fold change)
**Tooze et al.** (77)	**CLL** (PB; n=36) **SLL** (PB, BM, LN; n=24) **HD** (PB; CD20^+^CD5^+^ B cells; n=10)	FACS	CCR7 surface levels are lower in CLL than in SLL (1.6 fold change). CCR7 surface levels are lower in CLL than in CD5^+^CD20^+^ cells (2 fold change).
**Patrussi et al.** (66)	**CLL** (PB; n=42) **HD** (PB; n=18)	FACS IB/IF	High CCR7 surface levels in M and UM CLL were hypothesized to be a consequence of a high CCR7 recycling rate. Defects in p66Shc expression promoted this rapid turnover.

BM, bone marrow; B/R, Binet or Rai clinical staging; CLL, chronic lymphocytic leukemia; DCs, dendritic cells; FACS, fluorescence-activated cell sorting by flow cytometry; GEP, gene expression profiling; HA, hyaluronic acid; HD, healthy donor; IB, immunoblotting; IF, immunofluorescence; immunoglobulin; LN, lymph node; M/UM, IGHV mutated or un-mutated; MIF, mean intensity of fluorescence; MIFR, mean intensity of fluorescence relative to control; PB, peripheral blood; SLL, small lymphocytic lymphoma; SNP, single nucleotide polymorphism; TEM, transendothelial migration; tris12, trisomy in chromosome 12; UT/T, untreated or treated; VH, variable region in the Ig heavy chain; WBC, white blood cells.

In several reports, CCR7 expression and/or functionality has been associated with higher LN involvement and staging but no report directly associated this receptor, or its migratory response, with CLL patient survival (14, 42, 48, 49, 51, 53, 63, 78). Nonetheless, in other blood cancers such as diffuse large B-cell lymphoma (DLBCL) or T cell prolymphocytic leukemia (T-PLL) a clear correlation was found between CCR7 expression levels at diagnosis and OS (79, 80). Moreover, indirect evidence suggests a role of CCR7 in shortening lifespan in CLL. High levels of IκBα, a known repressor of NF-κB-mediated transcription of CCR7, correlated to extended OS in CLL (67). Similarly, *NOTCH1* mutations inducing a higher phosphorylation of the signal transducer and activator of transcription 3 (STAT-3) factor, and subsequent higher expression of CCR7, showed clinical characteristics of aggressive disease in a retrospective analysis on a cohort of 113 *NOTCH1*-mutated CLL patients (65). Moreover, serum levels for CCL19 are greater in CLL patients than in age-matched healthy subjects and those. Related to CCR7 ligands, serum levels for CCL19 were comparable between CLL patients and age-matched healthy subjects (81). However, within the CLL cohort, higher CCL19 levels independently associated with shorter survival. Moreover, when CCL19 was clustered along with other cytokines such as CCL3, CCL4, CXCL9, CXCL10, CXCL11, IFNγ, IL-5, and IL-12, this group of soluble factors was also found as an independent prognostic indicator of aggressive disease (time-to-first-treatment) and OS when compared to other clusters of cytokines (81). Therefore, it is likely that this group of cytokines provides a cytokine milieu that favors survival and growth to CLL cells.

### Mechanisms Underlying CCR7 Expression in CLL

In CLL, the key factors involved in the over-expression of CCR7 still remain to be uncovered. Nonetheless, recent evidence supports the hypothesis that both the inner genetic background of leukemic cells and/or environment factors facilitate CCR7 over-expression. To our knowledge, no potential *CCR7* gene mutations affecting transcription have been reported in CLL but one single-nucleotide polymorphism (SNP) in this gene was strongly associated with CLL risk (71, 82). Enjuanes et al. (81) found that the minor allele in the SNP rs3136687 (intron 1) resulted in a protective effect for the risk of CLL, although no CCR7 expression differences were observed for *such* allelic variants. This lack of association between *CCR7* genetic variants and CCR7 over-expression suggests the presence of alternative genotypes affecting other proteins that ultimately determine different signaling pathways controlling *CCR7* gene transcription and/or surface protein expression. In this regard, patient-associated DNA hypomethylation of the transcription factor NFATC1 (nuclear factor of activated T cells 1), a down-stream effector of the BCR, selectively facilitates expression of CCR7 (but not of other chemokines receptors such as CXCR4) and CCR7-induced migratory responses of CLL cells (76, 83). Other transcription factors known to regulate expression of CCR7 are NF-κB and activator protein 1 (AP-1); the latter is known to interact with NFAT protein (67, 84–86). NF-κB proteins are present in the cytoplasm bound to IκBα proteins, which are inhibitory molecules that sequester NF-κB dimers in the cytoplasm. Once BCR gets activated in CLL, IκBα is phosphorylated, ubiquitinated, and degraded in the proteasome. This facilitates NF-κB translocation to the nucleus, making the transcription of its target genes possible, including *CCR7* (67). In addition, BCR signaling in primary CLL cells through ZAP70 up-regulated *CCR7 via* an extracellular signal-regulated kinase (ERK)-1/2-dependent mechanism (59) although similar results were not seen in ZAP-70-overexpressing OSU-CLL cell line (87) and a recent study showed no regulation of CCR7 protein levels in CLL cells after IgM and IgD stimulation while CXCR4 and CXCR5 were down-regulated (64). Whatever the reason underlying those different outcomes are, these studies clearly show that BCR-activating environment factors might orchestrate the up-regulation of CCR7 in CLL as they do in normal B cells where CCR7 expression, following engagement of the BCR, is augmented through BCR-BTK signaling (88). This up-regulation facilitates T-B cells interaction by guiding B cells from the follicle to the border between the B and T cell zones in LN thanks to pre-established CCR7 ligand chemotactic pathways (89, 90). In this areas, B-T cell cross-talk is regulated by CD40-CD40L(CD154) receptors, another environment interaction known to up-regulate CCR7 expression in CLL, B-cell precursor acute lymphoblastic leukemia (BCP-ALL), or myeloid leukemia-derived DCs (74, 91–93). Accordingly, in CLL the activation of the CCR7 transcription factor NF-κB takes place after induction by CD40-CD40L ligation (94).

Another receptor implicated in sensing environment factors which has been shown to regulate CCR7 in T cell malignancies is the NOTCH1 transmembrane protein (95). In CLL, Arruga et al. associated *NOTCH1* mutations (which are found in ~10% of patients at diagnosis, ~20% of r/r patients, and ~30% in Richter’s syndrome) with STAT3-mediated CCR7 over-expression (65). This transcription factor is activated by mitogen-activated protein kinases (MAPKs) and directly regulates *CCR7* gene expression (96). In wild-type *NOTCH1* CLL cells, NOTCH intracellular domain (NICD) controls promoter methylation of the dual specificity protein phosphatase 22 (*DUSP22*) tumor suppressor gene that encodes a phosphatase that inactivates MAPKs, including c-jun N-term kinase (JNK) and p38, and dephosphorylates STAT3 (65). In a first step, NICD binds to the recombination signal binding protein RBPJk, which is bound to histone deacetylase 1 (HDAC1) in a heterodimeric repressor complex. Then, free HDAC1 binds and stabilize DNA methyltransferase 3A (DNMT3A), promoting DNMT3A activity and consequently the methylation of *DUSP22* promoter. Therefore, NOTCH1 mutations leading to constitutive activation of the NICD down-regulate DUSP22 levels, increasing MAPK and STAT3 activation which result in increased CCR7 levels. This mechanism was confirmed in a cohort of 113 CLL patients (65). Those patients with a NOTCH1-mutated clone showed significant hypermethylation of *DUSP22* with lower mRNA and protein levels of DUSP22, higher phosphorylation of STAT3 and expression of CCR7 and active chemotaxis to CCL19. Accordingly, patients with molecular or clinical characteristics of aggressive disease displayed significantly lower DUSP22 levels. Remarkably, another STAT family member, STAT-4 which is profoundly reduced in CLL cells (97) was implicated in *in vivo* down-regulation of CCR7 in T_H_ cells (98).

The scavenger receptor CD5 is another receptor implicated in the over-expression of CCR7 (99). Since this hallmark CLL phenotype marker is up-regulated in CLL cells, it is thought that increased CD5 signaling is another cause of differential CCR7 surface levels between normal and CLL B cells. Other studies suggest that *CCR7* gene is a reactive oxygen species (ROS)-responsive gene in B cells. Under normal conditions this gene is negatively controlled by the ROS-elevating activity of p66Shc, a cytoplasmatic pro-apoptotic protein, member of the Shc family of protein adaptors, and normally expressed in healthy B cells (42, 60, 66, 100). In CLL, the abnormal CCR7 surface levels were shown to be a consequence of the presence of ROS and a concomitant defect of p66Shc, which is also the cause of a rapid recycling of cell membrane CCR7 thus helping to maintain abnormal elevated membrane levels (42, 60, 66, 101). Curiously, the implication of a pro-apoptotic p66Shc protein in controlling CCR7 is not the only event of the apoptotic machinery involved in the regulation of this receptor. For example, some anti-apoptotic proteins such as Bcl-2 have been correlated with increased CCR7 expression in other tumor diseases and in non-tumor CD8^+^ T cells (102, 103). In these cases, a positive loop CCR7-Bcl2 promotes expression of the anti-apoptotic protein while Bcl2-signalling favors CCR7 production. Whether similar positive loops can be found in CLL is still unknown. However we do know that in CLL, activation of NF-κB after CD40-CD154 ligation, or activation of NFATC1 transcription factor, the downstream effector of BCR, results in both expression of CCR7 and Bcl2, therefore both events seem related to one shared former event (67, 76, 104, 105). Finally, it is worth noting that in solid cancers, such as breast cancer, a differential CCR7 expression between different histologic subtypes is determined by tumor microenvironment factors (e.g. hormonal, inflammatory, and growth stimulating) which may disable migration of CCR7-expressing tumor cells towards CCL21 and, consequently, the presence of LN migrations (106). Therefore, we cannot exclude finding similar requirements within the LN microenvironment for different CLL clones featured by differential combinations of adverse prognostic factors.

### CCR7-Induced Chemotaxis in CLL

It is known that levels of chemokine receptors do not always correlate with a higher migrative capacity (107). Instead, in CLL, the CCR7 surface over-expression does correlate with enhanced migration in response to CCR7 ligands, with this chemotactic response being more effective in CLL cells than in normal B cells (42, 48, 49, 55). Migration in CLL is likely mediated through a variety of downstream effectors, such as Rho, ERK and PI3K kinases (55, 59). These are assisted by several clonal factors that potentiate migratory responses favoring clinical lymphadenopathy and poor outcome, including the presence of certain adverse prognostic factors such as CD38, ZAP70, CD49d, trisomy 12, *NOTCH1* mutations, or un-mutated *IGHV* (42, 48, 49, 51, 53, 58–61, 64, 65, 75, 87, 108–111).

It is worth noting that CLL cells also express high levels of CXCR4 and CXCR5 (49, 112, 113). Despite this, leukemic CLL cells have a preferential *in vitro* migration towards CCR7 ligands. This situation contrasts with chemotaxis of normal B cells where CXCR4 and CXCR5 ligands are more effective than CCL19 or CCL21 (49). In this regard, a recent study by S. McHeik et al. demonstrated that expression of CCR7 in B cells selectively inactivated CXCR4, impairing migration towards its ligand CXCL12, and facilitating emigration from BM to PB (114). Mechanistically, up-regulation of CCR7 favors the formation of CXCR4-CCR7 heterodimers, thus acting as a selective endogenous allosteric modulator of CXCR4 that impairs its ability to activate certain G-protein complexes. These results may explain our observation in CLL migration (49) since up-regulation of CCR7 in CLL cells could favor the formation of CXCR4-CCR7 heterodimers thus reducing migration towards CXCL12. Nonetheless, we still have no data corroborating the presence of such regulatory heterodimers in CLL.

Finally, CCR7 not only directs migration of leukemic cells in CLL but, in addition, it is needed for migration of non-tumor subsets such as T cells. T cells from CLL patients and healthy donors have comparable expression of CCR7 and CXCR4 surface levels (52, 115), although CLL-associated T cells are less responsive to CXCL12, CCL21 and CCL19, except in T cells from patients with ZAP-70-expressing leukemic clones where migratory responses where similar to normal T cells (115).

### CCR7 and CLL Homing Into the LN

Emigration of normal T and B lymphocytes from the blood to LN is a well-defined multi-step process in normal lymphocyte trafficking, termed “homing”. Tethering and rolling of lymphocytes on HEVs mediated by selectins, chemokine receptor–induced integrin activation, integrin-dependent firm arrest on HEVs, and trans-endothelial migration are processes strongly dependent on CCR7 (31). Similarly, since the seminal work by Till et al. reported CCR7 as the main receptor involved in CLL cells entry into the LNs (48), there is much evidence to support that CCR7 takes over these processes which are exploited by CLL cells to enter LN (14, 61, 70, 72, 87, 116). In line, prominent HEVs with high amounts of CCL21 and CCL19 are found in CLL nodes (48, 74, 117).

For effective CLL cell homing, chemokine-mediated activation of the integrins lymphocyte function–associated antigen (LFA-1) and VLA-4 are key events needed for the initiation of shear-resistant arrest of B cells to endothelium (48, 87, 116, 118). Normally, CLL cells express low levels of these integrins, and thus few CLL clones enter the LN but some CLL clones with trisomy 12 or ZAP70 are associated with high levels of LFA-1 and VLA-4, allowing their entry into lymphoid organs. Accordingly, patients with trisomy 12 or ZAP-70 expression may show exacerbated lymphadenopathy (14, 58, 87, 111, 119, 120) but the molecular mechanisms underlying this clinical association have been largely unknown until recent works by Legler´s laboratory. The group identified a critical role for ZAP-70 in CCL21/CCR7-mediated, Src-dependent clustering and inside-out activation of the integrins VLA-4 and LFA-1 that facilitates CLL cell arrest on endothelial cells. ZAP-70 expression, however, seemed dispensable for other processes, such as migration velocity, chemokine-mediated crawling and diapedesis (87). Since ZAP-70 associates with *IGHV* mutational status (11, 12, 121, 122), it is reasonable to link CCR7/ZAP-70-induced homing to the un-mutated clones which have enhanced CCR7-triggered migration when compared to mutated ones (42, 64, 75). Mechanistically, CCR7 activation autophosphorylates Src kinase which in turn phosphorylates CCR7 at tyrosine 155 within the cytoplasmatic DRY motif of CCR7, providing a binding site for the Src homology 2 (SH2) domain containing signaling molecules (123). Then both SH2 domains of ZAP-70 directly interact with phosphorylated CCR7 and ZAP-70 promotes LFA-1 clustering which is essential for cell adhesion to intracellular adhesion molecule 1 (ICAM-1) on endothelial cells under physiologic flow conditions. Since the impact of ZAP70 is quantitative and not qualitative, Legler and colleagues propose that, because more cells arrest on CCL21-presenting endothelium, ZAP-70 expression enhances the chance of individual CLL cells to extravasate and thereby contributes to the accumulation of CLL cells in lymphoid tissues.

Despite the studies that strongly support the CCR7 axis as a main player in LN homing of CLL cells, some other chemokine receptors might also contribute to the characteristic disseminated lymphadenopathy in CLL, including CXCR3, CXCR4, or CXCR5 (48–50, 112, 124–126). Accordingly, normal B cells can exploit signaling mediated by CCR7, CXCR4, and CXCR5 to induce integrin-mediated arrest on HEVs and homing (127–130). Even though, only CCR7 seems relevant for LN homing as demonstrated in several *in vivo* models where CCR7 over-expression or genetic and pharmacology targeting impacted in the entry of CCR7-expressing cells into the LN (33–35, 62, 131, 132). In addition, CLL nodes have a disrupted architecture featured by the lack of follicles, which are the main source of CXCR5 cognate ligand, the chemokine CXCL13 (133, 134), and the chemokine CXCL12, the ligand of CXCR4, was not found in HEVs from CLL nodes (48). Taken together, these data suggest that CXCR4 and CXCR5 are not as relevant as CCR7 in CLL cell homing to LN. In agreement herewith, CLL cells transmigration through endothelium is more effective upon stimulation with CCL19 than with CXCL12 or CXCL13 (72). Under shear flow conditions, immortalized or primary CLL cells with trisomy 12 (associated to high levels of LFA-1 and VLA-4) significantly arrested to endothelial cells or VCAM/ICAM-1 coatings in the presence of CCL21, but not of CXCL12, since only the CCR7 ligand was capable to induce inside-out VLA-4 conformational changes as demonstrated in real-time kinetic assays (14, 87). Notably, similar results were obtained with CLL cells lacking trisomy 12. In another study, it was proposed that CCR7 might guide preferentially the homing of antigen-stimulated CLL cells since IgM activation selectively reduced migration of CLL cells towards CXCL12, but not CCL21 (64).

These arguments, substantiating the prevailing role of CCR7 in LN homing, rather than excluding the importance of CXCR5 or CXCR4 in the pathophysiology of CLL indicate that these receptors might act as synergistic, accessory molecules for CCR7 which may alter the global outcome in the process of LN homing. For example, in primary T cells and in one human Burkitt’s lymphoma cell line the exposure to CXCL12 potentiated *in vitro* transendothelial migration towards CCL19/CCL21, and also increased CCR7-dependent recruitment of T cells into LN *in vivo* (135, 136). On the other hand, CXCR4 might promote LN homing in an indirect manner, for example, favoring relocation of CLL from BM to the LN. In this respect, up-regulation of CCR7 in normal B cells selectively inactivates CXCR4 whereas mature B cells from CCR7^-/-^ mice display higher responsiveness to CXCL12 and improved retention in the BM (114). Accordingly, CXCR4 is the main receptor driving homing of CLL cells to BM where stromal cells provide protection from spontaneous or drug-induced apoptosis (51, 113, 137). Nevertheless, in certain CLL clones, such in trisomy-12-positive cells, CXCR4 expression is decreased and BM homing barely reliant on CXCL12-induced signals, despite a fully functional CXCR4 receptor in chemotaxis assays (14). Since these cells retained the ability to activate VLA-4 and to arrest on VCAM-1 in CCL21-stimulated CLL cells, one can propose a biased extravasation into LN tissues with pathophysiological consequences.

### CCR7 and LN Microenvironments in CLL

The survival of CLL cells is not a cell-autonomous, genetically determined process (138–141). Instead, CLL cells strictly depend on a permissive microenvironment that supports their survival and proliferation and consequently drives disease progression (2–4). CLL cells not only take advantage of CCR7 to home into the LN but also use this receptor for a correct positioning within the LN tissue as a result of the chemotactic routes created by stromal cells, which secrete CCR7 ligands (48, 74). In this regard, Höpken et al. demonstrated that genetic deletion of CCR7 in the syngeneic Eµ-Myc mouse lymphoma model was sufficient to exclude CCR7-deficient lymphoma cells from the T cell zone whereas wild-type lymphoma cells lodged to the stroma in close proximity to CD40L-expressing CD4^+^ T cells, DCs, and gp38^+^ fibroblastic reticular cells (FRCs), these latter providers of CCR7 ligands and the anti-apoptotic Indian hedgehog protein (Ihh) (34, 142). In turn, the lymphoma cells themselves secreted lymphotoxin through which they stimulated lymphotoxin-β-receptor–expressing FRCs. This crosstalk was necessary for the creation and preservation of protective niches in LN and spleen as demonstrated *in vivo* in adoptive transfer experiments since lack of CCR7 delayed disease onset and tumor burden (34, 142). Therefore, CCR7 enables distribution to different microenvironments where malignant cells may interact with supportive stroma (adhesion molecules and cells) and intra-tumor vasculature, crosstalk with CD40L-expressing cells, DCs, macrophages, and other tumor cells, recognize cognate antigens which triggers BCR signaling, and are exposed to soluble trophic factors: e.g. IL-7, IL-8, indoleamine 2,3-dioxygenase, CXCL12, CXCL13 (2–6, 34, 113, 140, 143–151). As a result, chemokines such as CCL3, CCL4, CCL19, CCL21, CCL22, CXCL12, CXCL13, are produced and a cycle of recruitment and proliferation begins in both CLL and accessory cells (147, 151–154). For example, recruited CCR7-expressing DCs in the LN may help further entry of tumor and other accessory cells directly through the production of CCL19 (131), or indirectly by promoting the secretion of vascular endothelial growth factor (VEGF) and CCL21 by FRCs (131, 155–157). The final outcome is a DC-induced vascular remodeling and generation of chemotactic cues for additional leukemic and accessory cells. Nonetheless, it should be kept in mind that increasing the presence of CCR7-expressing immune cells does not always associate with poor prognosis. The expression of CCL21 and endothelium proteins such as peripheral node addressin in LN-like vasculature found within solid tumors helps with anti-tumor T cell infiltration and positive prognosis in murine models (158). Unfortunately, similar studies showing a correlation between prognosis and the presence of CCR7-expressing infiltrating T-lymphocytes are still lacking in CLL. Finally, soluble CCL19/CCL21 also promote CCR7-induced survival of CLL cells through mechanisms not found in normal B cells. For example, CCL19 and CXCL13 cooperative signaling contributes to resistance to TNF-α-mediated apoptosis through up-regulation of paternally expressed gene 10 (*PEG10*) which stabilizes caspase-3 and caspase-8 (68, 78). Additional roles for CCR7-induced CLL cell survival have been described through MAP-kinases and PI3K-AKT signaling pathways upon binding of both cognate ligands (55, 69).

The knowledge about the mechanisms underlying the CCR7-guided interstitial CLL cell migration supports the idea that stop/go signals mediated by CCR7 in CLL are unique to this condition rather than resembling the well-known behavior of normal CCR7-expressing lymphocytes. In this regard, since CCR7 expression remains high within the LN and PCs (42) it is likely that CCR7 guides CLL cells to different niches assisted by other molecules. For example, interaction of wnt5 (A and B) or cordon-blue protein-like 1 (COBLL1) proteins with the receptor tyrosine kinase-like orphan receptor 1 (ROR1), a transmembrane receptor upregulated in CLL, activated pathways controlling cell polarity and migration. Specifically, ROR-1 activation increased basal migration and attenuated motility and chemotaxis toward CCL19 (159, 160) suggesting a role of these axes in fine-tuning CLL movement along CCL19 gradients and shutting migration off once the right niche is found. Interestingly, in non-tumor cells, COBLL1 expression was also found to be higher in GC B cells than naïve and memory subsets, where a marked reduction in CCR7 expression is needed to facilitate CXCR5-guided access to the GC (129, 161, 162).

The CC chemokine receptor-like 2 (CCRL2, also known as CRAM) is also aberrantly over-expressed in CLL B cells and it was proposed as a bystander molecule regulating CCR7-induced migration along CCL19 gradients (73), likely towards niches where the cognate antigen is found as suggested by the fact that CCRL2 is down-regulated upon IgD- or IgM-induced stimulation of BCR in CLL cells (64). However, the scavenging properties of CCRL2 in CLL are a matter of debate. In endothelial cells, recent evidence discards the binding of CCRL2 to CCL19 (among many other chemokines), the subsequent receptor internalization, and the ability to scavenge CCL19 (163, 164) precluding, therefore, its formerly proposed role as a shaper of CCL19 gradients. Instead, CCRL2 binds the non-chemokine chemotactic factor chemerin and presents it to cells expressing the chemokine-like receptor 1 (CMKLR1), the functional chemerin receptor (165). Since CMKLR1 is expressed in plasmacytoid DCs and endothelial cells it is tempting to speculate that the biological function of the axis CCRL2-chemerin-CMLKR1 in CLL has to do with facilitating interactions with these accessory cell types although future studies addressing this hypothesis are required to fully elucidate the mechanism by which CCRL2 regulates CLL cell functions.

Another peculiarity in CCR7-directed interstitial migration of CLL cells can be found in the important role attributed to the Cdc42-interacting protein 4 (CIP4), which is specifically overexpressed in CLL when compared with normal B cells or other subtypes of B-cell malignancies (166). For CCL19-driven directional cell steering and chemotaxis, CLL cells depend on CIP4 to modulate the Wiskott-Aldrich syndrome protein (WASP), p21-activated kinase 1 (PAK1), and p38 MAPK pathways to control the assembly of highly structured actin-rich protrusions, including lamellipodia. Remarkably all these factors (wnt-5A, wnt-5B, COBLL1, and CIP-4) are overexpressed in un-mutated *IGHV* CLL and are associated with poor outcome and nodal involvement. Once again, it seems reasonable that un-mutated or mutated *IGHV* clones interpret gradients of CCR7 ligands differently with the contribution of BCR downstream signaling proteins, such as ZAP-70 which has already been marked as an enhancer of BCR signaling and CCR7-mediated adhesion (61, 87). This differential behavior between mutated and un-mutated *IGHV* clones is not new. It is long known that un-mutated CLL shows a sustained interaction of competent BCR with low-affinity self-Ags, resulting in a higher proliferative rate, and only these clones usually respond to IgM stimulation (167–169). Similarly, IgM activation reduced migration of CLL cells towards CXCL12, but not CCL21, whereas IgD activation predominantly impacted on CCL21 but not CXCL12-mediated chemotaxis (64). This indicates a preferential role of CCR7 for migration of antigen-stimulated CLL cells within the lymphoid microenvironment.

Stromal proteins are also relevant players assisting CCR7 in the positioning of CLL cells close to CD40L-expressing cells, mainly CD4^+^ T_H_ cells. According to the model proposed by Hartmann et al. (74), unstimulated CLL cells use the nodal reticular network, simultaneously presenting hyaluronan (HA) and CCL21, as a guiding structure for their interstitial migration. The HA triggers a robust, random CCL21-induced motility of resting CLL cells until they encounter autologous T cells. If CLL cells get activated *via* CD40–CD40L signaling, then N-linked glycosylations of CD44 take place (particularly associated with the variant isoform CD44v6) and this glycoprotein strongly binds to HA causing CLL cells to stop migrating and instead tightly adhere to HA-bearing stromal cells. This strong CD44–HA–dependent adhesion facilitates cell division by retaining CLL cells in close proximity to CD4^+^ T cells in PCs, providing survival and proliferation cues: for example, T cell–derived interleukins (e.g. IL-4) (2–4). Interestingly, Hartmann and colleagues have demonstrated that this restriction to CCL21-induced motility is only based on physical blocking interactions since activated cells retain their migratory response towards CCL21 (74). Therefore, this mechanism to modulate CLL migration seems to override the continuous high, functional expression of CCR7 in the leukemic cells. Moreover, since CLL clones with unmutated *IGHV* present overexpression of CD44 (75), this proposed mechanism contributes to the enhanced retention of these clones within the LN by mediating adhesion to HA following CD40-CD40L engagement.

### CCR7 and Immune Tolerance in the LN

As further reviewed elsewhere (2–6), several studies suggest that CLL cells are not passive players within the tumor microenvironment (TME). Instead they actively modify it. We have already addressed their role in producing chemokines and cytokines that grant the access and/or promote proliferation of accessory gp38^+^ FRCs, DCs or CD40L-expressing T cells. In addition, CLL cells have the capacity to induce specific changes in myeloid and plasmacytoid DCs (170, 171), and T cells (172, 173) that can alter T cell immunological recognition and function resulting in an impaired immune response to the leukemia. Another mechanism that may contribute to creating a tolerant microenvironment for CLL is the presence of regulatory cells such as T_REG_ and the myeloid-derived suppressor cells (MDSCs). Both cell types have elevated frequencies in patients with CLL which associate with active disease (174–182) and, remarkably, both subsets express CCR7. Notably, this receptor is needed to enter the LN and other TME where CCR7 ligands are produced and where suppressive cells expand defeating antitumor immunity through heterogeneous mechanisms that include direct contact or the production of soluble factors such as IL-10 and TGF-β (183, 184). Indeed, in CLL the proportion of CCR7^+^ naïve natural T_REG_ is increased compared to healthy controls and within the overall T_REG_ population. Despite this population being characterized by augmented co-expression CD39, a molecule closely associated with suppressive activity in T_REG_, their suppressive activity (tested in 5 CLL patients) was not superior to other T_REG_ subsets (178). This result is not something unexpected since CCR7 expression is not needed for suppressive activity in T_REG_ (183) but for homing into LN where they respond to antigen and get armed performing their immunosuppressive activity. Without this step they still home to the peripheral tissues but cannot regulate (185–187). Nevertheless, further work on CCR7^+^ T_REG_ activity in CLL is needed in more samples to shed light on this aspect since patients could benefit from a therapeutic intervention consisting in the depletion of these suppressive cells. In other conditions, this therapeutic venue has been demonstrated to be effective in pre-clinical models of aggressive tumors (188, 189) and in clinical trials with mogamulizumab, an anti-CCR4 therapeutic mAb which selectively decreased the fraction of CCR4^+^ T_REG_ and associated with better clinical outcome in T cell malignancies and melanoma (190, 191).

### CCR7 Prolongs Residency of CLL Cells in LN Protective Niches

Besides its role in homing into SLOs, interstitial positioning, and survival, it has long been known that CCR7 is also crucial in the egress of lymphocytes from LN to PB. As shown in CCR7-deficient mice, absence of CCR7 forces a rapid egress from the LN, quicker than wild-type cells, whereas over-expression of this receptor retarded this process (192). Therefore, CCR7 expression and induced signaling pathways prolong the stay of lymphocytes in LN. Once lymphocytes find their cognate antigen, get activated, and undergo several divisions, CCR7 expression is progressively shut off (30, 192). CCL19-triggered signaling promotes down-modulation of CCR7 and a balanced up-regulation in the transcription of the counteracting sphingosine-1-phosphate receptor (S1P1) plays a crucial role in regulating the egress of T- and B cells from LN toward S1P1 rich circulatory fluids (blood and lymph), overcoming the retention signals provided by CCR7 (193).

In CLL, the work of Baldari et al. suggests that over-expression of CCR7, together with the concomitant low expression of egress S1P1, results in an altered balance that contributes to a prolonged residency of CLL cells in protective niches and subsequent lymphadenopathy (42, 60, 66, 100, 101). According to these authors, high CCR7 recycling rates in CLL cells seem to be one of the main contributors to receptor over-expression, with un-mutated CLL cells showing the highest turn-over and the more defective S1P1 expression. Mechanistically, deficiency of p66shc contributes to this process in two different ways. First, lack of p66Shc protein is the cause of CCR7 up-regulation and S1P1 down-modulation in CLL cells since the genes encoding both receptors are controlled in opposite directions by the ROS-elevating (pro-oxidant) activity of p66Shc (60, 100). Second, p66Shc inhibits the Ca^2+^-dependent activation of the phosphatase type 2B (PP2B, aka calcineurin) which dephosphorylates serine residues of CCR7, a step needed to release CCR7 from β-arrestin in early Rab5^+^ endosomes allowing transit to Rab11^+^ recycling endosomes (42, 66). The cause of p66shc deficiency in CLL remains unknown, however p66Shc expression is regulated by STAT-4, which is profoundly reduced in CLL cells (97). Together these findings are highly relevant as they uncover a novel regulatory mechanism for CCR7 in B cells where the receptor was thought not to be completely needed to balance stay/egress from lymphoid tissues (192). p66Shc deficiency does not impact S1P1 expression in T cells (60). Moreover, p66Shc deficiency seems highly specific to leukemic CLL B cells and more specifically in un-mutated *IGHV* CLL clones. However, it is important to keep in mind that other factors may contribute to the unbalanced CCR7/S1P1 signaling in CLL cells. For example, un-mutated *IGHV* cells show low levels of S1P1 signaling proteins such as dynamin-2 and G-protein α_i_  (75). In addition, un-mutated clones overexpress CD44. As already discussed, CD44 contributes to the retention of CLL cells in LN by mediating adhesion to HA following CD40-CD40L engagement, thus contributing, in and additional manner, to prolong residency of CLL cells within the LN (74, 75, 91).

## CCR7 as a Novel Therapeutic Target in CLL

The evidence presented here suggests that novel tools targeting CCR7 are appealing to displace cells from LN microenvironment, hitting, therefore, the “*Achilles’ heel*” of CLL. As such, the present authors and other teams demonstrated that CCR7 genetic deletion or pharmacological inhibition with anti-CCR7 monoclonal antibodies (mAb) reduced CCR7-triggered migration and homing into LN in both *in vitro* and *in vivo* pre-clinical models (34, 62) (52, 55, 63). Similarly, driving leukemic cells out of LN to induce “death by neglect” or forcing their apoptosis are the mechanism of action (MOA), respectively, of ibrutinib and venetoclax, the current standards-of-care (SOC) in CLL (21, 22, 194).

Recently, it was reported that allosteric antagonism by small molecules under clinical investigation interfered with CCL19-driven signaling (195). Thus this work confirmed a long-standing interest in active synthetic drugs to avoid entry of cancer cells into LN. Our laboratories’ work focused on antibodies which may provide several advantages over small molecules: selectivity, affinity, increased serum half-life and tumoricidal capacities (196, 197). Moreover, small molecules are unable to trigger host anti-tumor responses whereas therapeutic antibodies have provided such clinical benefits to cancer patients during the last decades (198, 199). We hypothesized that a higher efficacy at reducing LN tumor burden would be achieved by a neutralizing anti-CCR7 monoclonal antibody (mAb) able to immobilize cancer cells and to elicit cell killing (52, 62, 63). However, raising blocking antibodies against CCR7 and other CKR has been considered a challenging task due to the necessity of targeting specific epitopes involved in ligand binding, and to a high sequence homology between human and mouse CCR7 that impairs immunogenicity (197). In agreement herewith, few anti-CKRs are under study in pre-clinical or early clinical phases, and only one (mogamulizumab) has been approved for clinical use (197, 200–202). Fortunately, the advent of novel technologies including sophisticated purification techniques, synthesis of conformational peptides, genetic immunization techniques, production of CKR-containing liposomes or lipoparticles, or over-expression of receptors in viral particles (197, 202) has overcome cited limitations and the first therapeutic anti-CCR7 antibodies have been developed. Recently, Novartis has announced the beginning of a phase I trial with JBH492 an antibody targeting CCR7 (NCT042140704). Despite not being clear if this molecule blocks the target, the fact that it is linked to a cytotoxic molecule (antibody-drug-conjugate, ADC) indicates that killing tumor cells by means of the release of cytotoxic payloads seems its main MOA. In addition, Catapult Therapeutics disclosed first pre-clinical results of a novel humanized IgG1 antagonist (CAP-100) specifically developed for cancer therapy which will be evaluated in a first-in-human clinical trial (NCT04704323) in 2021 (203, 204). This antibody is featured by a unique dual MOA which relies on an effective combination of strong blocking and killing activities. This antibody neutralizes receptor-ligand interactions thus inhibiting CCR7-induced cell functions, such as the access of cancer cells to niches where CCR7 ligands are produced (e.g. LN or spleen). This way, tumor cells are forced to accumulate in the bloodstream where they become more accessible to indirect cell killing mediated by effector immune cells (ADCC, antibody-dependent cell-mediated cytotoxicity), to spontaneous apoptosis, or to other treatments. Targeting CCR7 is a plausible strategy not only as a monotherapy but may also contribute in a rational combination to enhance current standard-of-care treatments. For example, the use of neutralizing anti-CCR7 antibodies along with ibrutinib would synergistically target CCR7-induced integrin-mediated adhesion to lymphoid stroma (42, 61, 66), thus enhancing drug-promoted CLL cell mobilization from protective lymphoid niches into circulation. Additionally, the antibody would block recirculation and loops of LN homing. Ibrutinib would also interfere with CXCR4- and CXCR5-mediated signaling and with the production of chemokines (CXCL12, CXCL13, CCL19) by myeloid stroma cells (61, 151). Then, spontaneous death in cells deprived of critical microenvironment growth and survival signals will be supplemented by antibody-induced cell killing. Similarly, anti-CCR7 mAbs combined with PI3Kδ inhibitors such as Idelalisib (205), a selective inhibitor of the p110 delta isoform, might contribute to inhibition of integrin-mediated arrest of CLL cells on endothelial cells. In other combinations, anti-CCR7 antibodies would mainly interfere with homing into LN and with the following interstitial migration within this tissue, whereas adjuvant therapies, such as the Bcl-2 inhibitor venetoclax, would collaborate in inducing cell death. Interestingly, venetoclax is markedly less toxic towards CLL cells when co-cultured with activated T lymphocytes (206) therefore, anti-CCR7 therapies could reverse this situation by impairing the support of CLL cells by accessory T cells. Finally, it is worth mentioning that un-mutated CLL clones are preferentially retained in LN, where they are exposed to proliferative stimuli, suggesting that anti-CCR7 strategies may be particularly effective in un-mutated-CLL. In agreement with this prediction, clinical studies have shown that the overall response to ibrutinib or idelalisib is significantly higher in patients with un-mutated CLL compared with those with mutated clones (61, 205, 207–209).

## Safety of Anti-CCR7 Therapies in CLL

Today, the main concerns linked to the potential of CCR7 as a therapeutic target have to do with the fact that CCR7 is a critical molecule for inducing adaptative immune responses as well as for the generation of T_REG_ cells that control the development of self-reactive cells and subsequent auto-immunity. Although most of the knowledge on the involvement of CCR7 in these processes is derived from CCR7-deficient mice (33) and paucity of lymph-node T cells (plt) mice, a naturally occurring strain that carries an autosomal recessive deletion that contains the genes encoding CCL19 and CCL21-Ser (210, 211), the expression patterns of CCR7 in mice and humans are similar thus corroborating their use for the *in vivo* investigations of this CKR. From these models we learnt that lack of CCR7 signaling did not compromise life or life-span, and animals were not immuno-deficient in a strict sense. On the contrary, these phenotypes associated with retarded but effective T cell and B cell responses (33, 129, 212), especially in cases where a replicating antigen (viruses and some bacterial infections) was present (213–216). We also learnt that lack of CCR7 reduced LN homing of T_REG_ and their right positioning in the nodal environment hampering, therefore, central and peripheral tolerance (183, 217–220). In the long-term, CCR7-defficient mice were prone to develop generalized multi-organ autoimmunity (mainly in mucosa) although had a normal life span and did not suffer from clinically aggressive diseases. Finally, it is important to remark that also in CCR7-deficient mice autoimmunity is a multi-factorial process related to mice strain (genetic predisposition) and environmental factors (219–221) and that many phenotypes in CCR7-deficient animals are a consequence of immunity development within abnormal SLO microenvironments (222). Therefore, it is coherent to think that the immune responses of a patient treated with therapies targeting CCR7 will not fully mirror the outcomes of CCR7-deficient animals. In this sense, pre-clinical studies have shown selective effects of anti-CCR7 mAbs for CLL cells while sparing healthy counterparts, even at saturating concentrations of antibody (52, 63, 223, 224). In these studies only T_N_ and T_CM_ cells were impacted (though not entirely removed) while other CCR7-expressing cells such as DC or B cells were surprisingly not affected. We and others believe that this effect is likely due to a lower target density in non-tumor cells or to a lower affinity of these antibodies for CCR7 expressed in these cell types. These results lead to expect a low-to-mild associated immunosuppression in patients receiving therapeutic doses of an anti-CCR7 mAb. For example, it is likely that anti-CCR7 therapy could impair new immunization processes dependent on T_N_ cells, however it should not affect memory effector responses against infections (224). Similarly, CCR7-negative T_EFF_ and T_EM_ rather than CCR7-expressing naïve or T_CM_ are necessary to effective anti-tumor responses (225). Targeting CCR7 could also affect B cell homing during antigen-dependent and independent B cell differentiation; however, CCR7-deficient mice show splenic B cell responses upon bacterial challenge (129). This regard, normal B cells are less dependent on CCR7 than CLL cells for arrest on HEVs and homing (127, 133, 192) while B cell BM precursors and plasma cells lack CCR7 (49), thus suggesting that CCR7 therapy would not suppress B cell lymphopoiesis nor immunoglobulin secretory function (33, 129).

As previously indicated, T_REG_ is another cell type that might be affected by anti-CCR7 therapies. We have already seen how this subset is significantly increased in CLL patients and correlates with poorest clinical outcomes (174–178). However, whether targeting CCR7-expressing T_REG_ would be beneficial or deleterious for CLL patients is a controversial issue. By one hand, interfering T_REG_ functions is an undesired side-effect associated to the development of autoimmunity in CLL patients treated with the PI3Kδ inhibitor idelalisib (226). On the other hand, these frequent unwanted events linked to idelalisib were not as prevalent in mAb-based therapies with the discontinued anti-CD52 antibody alemtuzumab (which depletes pan-T cell populations, including T_REG_) (227) or with the anti-CCR4 antibody mogamulizumab (which removes CCR4^+^ T cell subsets, including T_REG_) (228). Finally, it is worth to indicate that anti-CCR7 therapy in pre-clinical syngeneic mouse models of cancer, autoimmunity, GVHD, or inflammation did not uncover un-wanted treatment-associated side effects (184, 223, 229) and CAP-100 toxicology studies in NHP did not reveal overt toxicities or autoimmune disease indicating tolerability of this novel therapy. In the coming months, first data in patients receiving a chronic administration of an anti-CCR7 mAb will shed light on these safety issues.

## Conclusions

**Figure 2:**
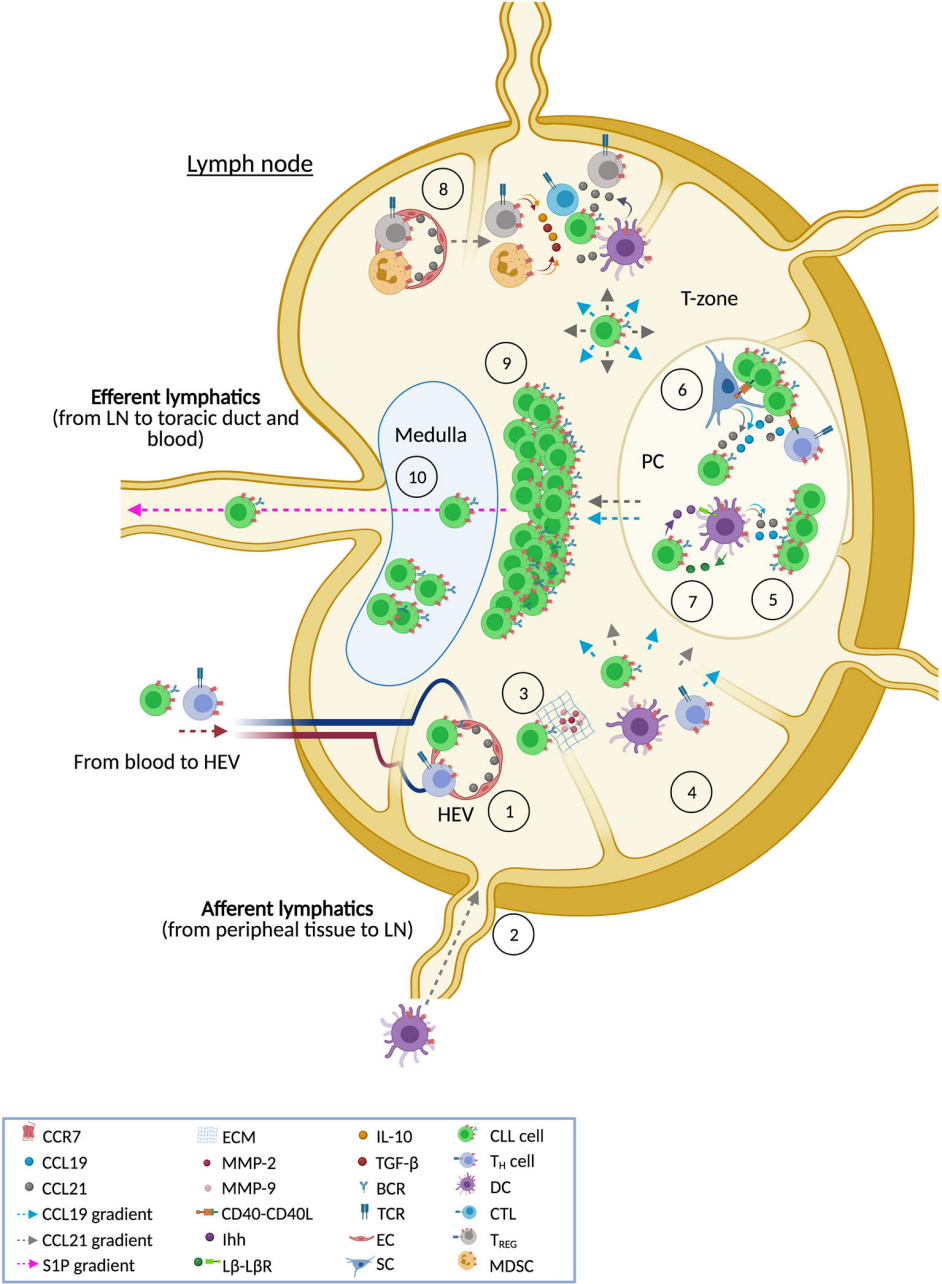
CCR7 and the lymph node in CLL. The figure shows the different ways in which CCR7 contributes to CLL pathobiology in the LN tissue. This receptor directs leukemic and accessory cells into the LN following CCL21 gradients that allow cells to across high endothelial venules (HEV) [1]. It is also likely that CCR7 might promote entry through a different gate, the afferent lymphatic vessels [2], although this last situation has not been reported yet in CLL cells. Both entry points are also used by accessory cells such as T cells and dendritic cells (DCs). When CLL cells get access through HEV, binding of CCL21 and subsequent CCR7 signaling promotes a more invasive phenotype, featured by enhanced production of matrix metalloproteases (MMP-2 and MMP-9) that degrade the extracellular matrix (ECM) [3]. This process facilitates trans-endothelial migration and the following interstitial migration within the LN tissue following CCL19 and CCL21 gradients favoring the right positioning of CLL cells within niches where accessory cells, stroma components, or soluble factors (e.g. cytokines and chemokines) are available [4]. Accessory and stromal cells are the main producers of CCR7 ligands thus facilitating the creation of chemotactic routes towards these niches. Similarly, some CCR7-expressing accessory cells can be directed by CCR7 ligands to these environments. Once CLL cells are driven to protective niches, such as proliferation centers (PC), tumor cells have access to CCL19 and CCL21 (which are produced by stromal cells and DCs) which rescue CLL cells from spontaneous or drug-induced apoptosis [5]. CLL cells also have access to BCR signaling and CD40-CD40L signaling [6] which regulate both CCR7 expression and chemotaxis in CLL cells further contributing to interstitial movement within the LN tissue. In the protective niches, CCR7 signaling in CLL cells is also involved in the secretion of trophic factors needed by accessory cells thus creating a positive feedback loop to preserve these tumor niches. For example, CLL cells themselves might preserve PC by means of secretion of lymphotoxin β (Lβ) which binds to Lβ-receptor in stromal cells and induces their differentiation into pro-tumor cells [7] which secrete Indian hedgehog protein (Ihh) triggering survival in malignant cells. Similarly, CLL cells can modulate activity of anti-tumor immunity through the recruitment of pro-tumor regulatory cells such as T_REG_ and myeloid-derived suppressor cells (MDSC); both subtypes characterized by expression of CCR7 which orchestrates their homing into the LN [8]. These suppressor cells inhibit anti-tumor effector cells (CTLs, NK cells, B cells, etc) through cell-cell interactions or well by creating a tolerant milieu enriched in IL-10 and TGFβ. As a result of all these described activities [5–8], CCR7 directly or indirectly promotes tumor growth in the T cell zone of the LN [9], contributing to the typical obliterated enlarged structure in CLL nodes. Moreover, CCR7 up-regulation in CLL cells (as a consequence of an aberrantly rapid recycling rate of the receptor) leads to an impaired up-regulation of S1P1, the receptor guiding the egress of immune cells trough S1P gradients towards the efferent lymphatic vessels. Therefore, CCR7 signaling retains CLL cell within the LN, increasing the residence time in protective niches thus contributing in an additional way to bulky disease in the LN [10].

The LN is the main hub for CLL where leukemic cells find proliferation and survival cues. Among the multiple altered signaling pathways found in CLL, the axis CCR7-CCL19/CCL21 is especially relevant (**Figure 2**). As revised here, CCR7 over-expression is a feature of CLL that has historically been reported but, until recently, we did not begin to understand the exact mechanisms underlying this process. Interesting novel findings suggest that altered receptor recycling pathways are involved in this up-regulation. Nonetheless, these abnormal processes at the protein levels could be just the tip of the ice-berg and additional studies addressing another mechanisms, such as genetic or epigenetic dysregulation, would be of great interest. The canonical view of CCR7 as a LN homing receptor of CLL cells has been up-dated thanks to studies revealing CCR7 as a key mediator of interstitial migration towards PCs and other locations where CLL cells cross-talk with accessory cells and have access to trophic factors which promote tumor growth and progression, including CCR7’s own ligands. The extensive knowledge arisen on the pathogenic roles of CCR7 in CLL cells contrasts with the scant studies focused on receptor-mediated functions in accessory cells during the different phases of niche colonization, preservation and progression. Fortunately, the advent of novel BCR inhibitors has indirectly provided first clues for a better understanding of the mechanisms through which CCR7 creates and preserves protective and tolerogenic milieus, prolongs CLL cell residency in these niches (contributing, therefore, to lymphadenopathy), and provides escape from therapeutic agents. Gathered together, this current extensive collection of evidence confirms CCR7 as key molecule in CLL and suggests, therefore, that tools targeting CCR7 are appealing as novel therapies in CLL. Today, two novel anti-CCR7 mAbs are facing clinical evaluation. Although both compounds have shown high activity in pre-clinical models, first-in-human studies need to solve several questions, beyond efficacy, that are linked to this kind of therapy such as the associated risk for impairing adaptative immunity and/or developing auto-immunity. If positive results are obtained in this clinical trials, we will likely witness to an exciting new age not only in CLL therapy but also in other diseases where CCR7 mediates deleterious roles.

## Author Contributions

CC-M, FT, JB, and CM-C contributed equally to the conception, design and writing of this manuscript. All authors contributed to the article and approved the submitted version.

## Conflict of Interest

CC-M is an employee of Catapult Therapeutics and of Immunological and Medical Products (IMMED S.L.), and a shareholder in this last company. FT declares that he is CEO and a shareholder in the same companies. CM-C is a consultant for IMMED S.L., who has a granted patent for the use of therapeutic antibodies targeting CCR7 in cancer and has received research funds from IMMED.S.L. and Catapult Therapeutics. She also holds shares in IMMED S.L. JB has served as a consultant for Abbvie, Acerta, Astra-Zeneca, Beigene, Catapult, Dynamo Therapeutics, Eli Lilly, Genentech/Roche, Gilead, Juno/Celgene/Bristol Myers Squibb, Kite, Loxo, MEI Pharma, Nextcea, Novartis, Octapharma, Pfizer, Pharmacyclics, Rigel, Sunesis, TG Therapeutics, Verastem; received honoraria from Janssen, received research funding from Gilead, Loxo, Sun, TG Therapeutics and Verastem; and served on data safety monitoring committees for Invectys.
